# Dietary Habits and Their Influence on the Microbiome and Mental Health in Adolescents

**DOI:** 10.3390/nu17091496

**Published:** 2025-04-29

**Authors:** Andreea Sălcudean, Dora-Mihaela Cîmpian, Ramona-Amina Popovici, Norina Forna, Diana-Mihaela Corodan-Comiati, Andreea-Bianca Sasu, Melania-Maria Cozma, Cristina-Raluca Bodo, Eduard-Cristian Enache, Mariana Păcurar, Ramona-Elena Crăciun, Alexandru Blidaru, Viorel Jinga, Maria-Dorina Pașca, Emese-Erika Lukacs, Mariana-Cornelia Tilinca, Elena-Gabriela Strete, Andrada-Ioana Crișan, Bianca-Eugenia Osz, Daniela-Lucia Muntean

**Affiliations:** 1 Department of Ethics and Social Sciences, George Emil Palade University of Medicine, Pharmacy, Science and Technology of Târgu Mureş, 540142 Târgu Mureş, Romania; andreea.salcudean@umfst.ro (A.S.); melaniacozma76@gmail.com (M.-M.C.); cristina.bodo@umfst.ro (C.-R.B.); mdpasca@yahoo.com (M.-D.P.); 2 Department of Management and Communication in Dental Medicine, Faculty of Dental Medicine, Victor Babes University of Medicine and Pharmacy of Timisoara, 300041 Timisoara, Romania; 3 Department of Implantology and Prosthetic Implant Rehabilitation, Faculty of Dental Medicine, “Grigore T. Popa” University of Medicine and Pharmacy, 700946 Iași, Romania; norina.forna@umfiasi.ro; 4 Psychiatry Clinic 1, County Clinical Hospital Mureș, 540136 Târgu Mureș, Romania; corodan.mihaela@yahoo.ro (D.-M.C.-C.); andreea_vinteler@yahoo.com (A.-B.S.); 5 Faculty of Medicine, George Emil Palade University of Medicine, Pharmacy, Science and Technology of Târgu Mureş, 540142 Târgu Mureş, Romania; enache.eduard-cristian@stud19.umfst.ro; 6 Orthodontic Department, Faculty of Dental Medicine, George Emil Palade University of Medicine Pharmacy, Science and Technology of Târgu Mureș, 540142 Târgu Mureș, Romania; marianapac@yahoo.com; 7 Association “Child and Childhood”, 540472 Târgu Mureş, Romania; r.craciun@yahoo.com; 8 Surgical Oncology Department, University of Medicine and Pharmacy “Carol Davila” Bucharest, 020021 Bucharest, Romania; alexandru.blidaru@umfcd.ro; 9 Department of Urology, University of Medicine and Pharmacy “Carol Davila” Bucharest, 020021 Bucharest, Romania; viorel.jinga@umfcd.ro; 10 Department of Psychiatry, George Emil Palade University of Medicine, Pharmacy, Science and Technology of Târgu Mureş, 540142 Târgu Mureș, Romania; emese.lukacs@umfst.ro (E.-E.L.); elena.buicu@umfst.ro (E.-G.S.); 11 Department of Internal Medicine I, Faculty of Medicine in English, George Emil Palade University of Medicine, Pharmacy, Science, and Technology of Târgu Mureş, 540142 Târgu Mureș, Romania; mariana.tilinca@umfst.ro; 12 Doctoral School, George Emil Palade University of Medicine, Pharmacy, Science and Technology of Târgu Mureș, 540142 Târgu Mureș, Romania; crisanandrada@yahoo.com; 13 Faculty of Pharmacy, George Emil Palade University of Medicine, Pharmacy, Science and Technology of Târgu Mureș, 540142 Târgu Mureș, Romania; bianca.osz@umfst.ro; 14 Department of Analytical Chemistry and Drug, George Emil Palade University of Medicine, Pharmacy, Science and Technology of Târgu Mureș, 540142 Târgu Mureș, Romania; daniela.muntean@umfst.ro

**Keywords:** gut–brain axis, microbiome, diet, adolescents, mental disorder, dysbiosis

## Abstract

Adolescence represents a critical developmental stage where diet, gut microorganisms, and mental health are strongly interconnected. The current literature evidences the bidirectional role between dietary habits and psychological well-being, which is mediated by the gut–brain axis. The purpose of this review is to highlight the importance of dietary habits in adolescence period and the impact of different food choices on microbiota and secondary on mental health. Gut microbiota plays a vital role in the synthesis of neurotransmitters such as serotonin, dopamine, noradrenaline, and metabolites like short-chain fatty acids (SCFAs). The disruption in the composition of microbiota is called dysbiosis, which has been associated with a systemic inflammation state and chronic stress. They contribute to the onset of psychiatric disorders including MDD, anxiety, ADHD, and autism. Diets with a high quantity of sugar and low fiber contribute to alteration of microbiota and poor mental health. Additionally, early-life stress, antibiotic usage, and chronic inflammation may alter bacterial communities, with long-term implications for adolescents mental health. Dietary interventions, including the intake of prebiotics, probiotics, SCFAs, and micronutrients could restore microbial balance and improve psychiatric symptoms. This literature review highlights the critical role of diet and gut microbiota for adolescent mental health and emphasizes the need for integrative strategies to promote psychological resilience through microbiome regulation.

## 1. Introduction

Adolescents’ dietary habits have the potential to significantly influence both gut microbiota composition and mental health. Numerous studies have investigated the implications of nutrition on human behavior and functioning. The relationship between diet and mental health is bidirectional [1]. Dietary changes may lead to psychological alterations, while certain mental health disturbances could also impact eating behaviors, suggesting an interdependent mechanism [1].

Current research shows that adolescents exposed to high levels of stress may experience alterations in their gut microbiome, which in turn may influence the severity of depressive symptoms [2]. A sedentary behavior, combined with a high intake of processed, sugar-rich foods, has been associated with increased rates of obesity among adolescents. This phenomenon is further linked to an increased prevalence of depressive symptoms, suggesting a detrimental impact on mental well-being. Inflammatory state and high cytokine production [3] have also been shown to negatively affect mental health outcomes [4].

For example, a study on adults with obesity revealed that a very low-calorie ketogenic diet (VLCKD) induced beneficial changes in gut microbiota composition, shifting it toward a more balanced and anti-inflammatory profile [5]. These microbial shifts may play a role in improving psychological and metabolic outcomes, highlighting the impact of dietary interventions on mental health [5].

Over the past decade, researchers have focused on the gut–brain axis. It has been well established that complex and bidirectional communication exists between the central nervous system (CNS) and the gastrointestinal tract, mediated through neural, endocrine, and inflammatory pathways [6]. The gut–brain axis involves interconnections among the following systems and structures: the central nervous system (CNS), the neuroendocrine and neuroimmune systems, the hypothalamic–pituitary–adrenal axis (HPA), the sympathetic and parasympathetic branches of the autonomic nervous system, the endocrine system, the enteric nervous system, and the vagus nerve [6,7,8].

The gut microbiota is responsible for the synthesis of several neurotransmitters that have an important role on CNS, including gamma-aminobutyric acid (GABA), dopamine, adrenaline, serotonin, and acetylcholine. It may also influence serotonin production by modulating plasma tryptophan levels [9]. Notably, studies have also indicated a reverse effect: elevated concentrations of certain neurotransmitters may promote the growth of pathogenic bacteria. For example, the neurotransmitter noradrenaline can facilitate the proliferation of *Klebsiella pneumoniae*, *Pseudomonas aeruginosa*, *Shigella sonnei*, *Staphylococcus aureus*, and *Escherichia coli* [7,10].

Furthermore, the scientific literature emphasizes the vital role of the gut microbiota in processes such as neurogenesis and myelination within the central nervous system (CNS) [11,12,13,14]. Emerging evidence suggests that the gut microbiome plays a fundamental role in the development of various psychiatric disorders in children, adolescents, and adults, including autism-spectrum disorders, depressive disorders, and anxiety disorders [15,16,17,18].

Chronic stress and psychological factors may influence the level of the stress hormone—cortisol [18]. Studies reveal that an increased level of cortisol may alter the composition of microbiota during childhood and adulthood [19,20,21].

This literature review highlights the critical role of diet and gut microbiota for adolescent mental health and emphasizes the need for integrative strategies to promote psychological resilience through microbiome regulation.

## 2. Methodology

This literature review was conducted using a narrative approach to explore current knowledge on the dietary patterns observed among adolescents and their impact on gut microbiota and mental health. A comprehensive search of electronic databases including PubMed and Google Scholar was performed using keywords such as “gut microbiota, intestinal flora, microbiome composition, microbiota, microbiome, dietary patterns, nutrition, eating behaviors, diet, eating habits, junk food, mental health, depression, anxiety, mood disorders, ADHD, autism, metabolic disorders, obesity, adolescents, adolescence, children, childhood, young people, teenagers, gut–brain-axis, inflammatory markers, neurotransmitters, serotonin, prebiotics, probiotics”.

Original preclinical and clinical research, review articles, and case reports were included. Articles published in English between 2010 and 2025 were considered. Research conducted before 2010 was excluded.

## 3. The Gut Microbiome: Fundamentals and Importance in Mental Well-Being

The microbiome, along with its composition and functional implications, has become an increasingly prominent area of scientific investigation over the past few decades [8]. Recent studies have emphasized the importance of intestinal microorganisms in maintaining human health. Thus, in recent decades, gut microbiome became an important marker in different physical and mental pathologies [9,22,23].

The human microbiome consists of diverse microorganisms—including bacteria, viruses, and fungi, which are distributed throughout the gastrointestinal tract (GIT) [7,24]. A healthy individual is typically colonized by approximately 10 to 100 trillion microbial cells. The structure and composition of these microbial colonies may vary significantly from person to person, and two different humans may possess entirely distinct microbiomes [25,26,27].

The colonization of the gut microbiota begins during birth, as the infant passes through the vaginal canal and becomes exposed to maternal flora. At the age of two, the child’s microbiota becomes diversified, primarily influenced by dietary factors. Thus, this early microbiota composition forms the base of the individual’s adult microbiome composition [26].

Several factors may influence the composition of the intestinal microbiota, including age, sex, mode of delivery, aging, diet, geographical location, genetic predisposition, medication usage, and stress factors [24,26,28]. Additionally, external behavioral factors such as smoking, alcohol consumption, and the consumption of psychoactive substances may influence microbiota composition, potentially leading to dysbiosis.

Overall, the gut microbiome consists of bacteria that are structured in phyla, classes, families, genera, species, and strains [29]. Approximately 90% of a healthy person intestinal microbiome is composed of commonly found phyla: Bacteroidetes, Actinobacteria, Proteobacteria, and Firmicutes [30,31,32,33,34,35]. The remaining 10% consists primarily of Fusobacteria and Verrucomicrobia [31]. These bacterial communities play a critical role in metabolism, leading to the production of essential metabolites such as vitamins, amino acids, short-chain fatty acids (SCFAs), lipopolysaccharides (LPS), and trimethylamine-N-oxide (TMAO) [26]. These bacterial metabolites have a significant role in the pathogenesis of major depressive disorders (MDDs) [36].

Specific microorganisms are directly involved in neurotransmitter production. For instance, *Candida*, *Enterococcus*, *Escherichia coli*, and *Streptococcus* species influence serotonin production [30]. The production of gamma-aminobutyric acid (GABA) is facilitated by *Lactobacillus* and *Bifidobacterium*, while dopamine synthesis is supported by Bacillus and Serratia species [30]. Fungi such as *Saccharomyces boulardii* contribute to the biosynthesis of noradrenaline (NA). These microorganisms are essential in the metabolic pathways responsible for generating neurotransmitter precursors [30].

The composition of gut microbiota, including the ratio between Firmicutes/Bacteroides, might be associated with several pathologies including mental disorders and obesity in childhood [37,38] and adulthood [16]. Some of the bacteria may have a protective role against mental disorders, while others are linked to the onset of these pathologies. Anxiety is associated with a decreased ratio between Firmicutes and Bacteroides species. Depression is related with an increased ratio between Bacteiroides/Firmicutes, characterized by an increased quantity of the Bacteiroides spectrum, and a depletion of *Blautia*, *Coproccocus*, and *Faecalibacterium* [39]. Autism-spectrum disorders are associated with a high level of *Clostridium boltae*, *Clostridium paraputri*, and *Clostridium perfringens* [16].

Recent studies found that there are important correlations between the metabolites of gut microbiota and psychiatric disorders. Short-chain fatty (SCF) acids like acetate, propionate, and butyrate are depleted in major depressive disorders [40].

Neurotransmitters produced directly or indirectly by the gut microbiota (serotonin, dopamine, GABA, norepinephrine) may have an impact on the emotional response and behavior through a modulatory mechanism on the central nervous system [41].

The bacterial metabolites and changes in the gut microbiome, called dysbiosis, cause inflammation and have a major impact on the onset of diabetes [42]. It is significantly important to understand this mechanism before the initiation of anti-diabetic medication and modification in diets. There are studies that suggest there is a possible connection between serotonin levels and its prediction for MDD in patients with diabetes [43,44].

Inflammatory bowel disease may be caused by an altered composition of intestinal bacteria. Furthermore, the chronic inflammation state maintains dysbiosis and influences levels of intestinal metabolism of neurotransmitters, thus having an important impact on psychiatric disorders’ onset [45]. Dysbiosis contributes to systemic inflammation onset which plays an important role in exacerbation of neuroinflammation state [46]. Neuroinflammation plays an important role in mental health disorders such as MDD [45,47,48].

## 4. Diet and Its Influence on the Microbiome

A nutritionally balanced diet ensures the host receives a complete profile of necessary nutrients and promotes the proliferation of beneficial gut microorganisms [49,50]. Dietary components have a significant impact on host physiology that extends beyond their nutritional value, primarily through interactions with the gastrointestinal microbiome. This intricate interplay also involves the immune system and the intestinal barrier, underscoring the central role of diet in both the pathogenesis and treatment of various diseases [51,52]. Understanding the significant impact of different diets on the microbiome is essential, as it allows us to make informed dietary choices that promote better metabolic and intestinal health. Moreover, it helps in specific diet-associated disorders that arise from suboptimal nutritional habits [53,54,55].

### 4.1. Dietary Fibers

Within the dynamic ecosystem of the gut, dietary fiber plays a vital role in fermentation and serves as a key nutritional source for gut bacteria. Research has demonstrated that a diet high in fiber has been linked to increased production of short-chain fatty acids (SCFAs). These SCFAs have been directly linked to the growth of beneficial bacterial strains, as well as to protective functions in maintaining gut-barrier integrity and regulating local gut inflammation [56]. Growing evidence highlights the positive impact of dietary fiber intake on overall health. In recent years, the underlying mechanisms have become clearer, emphasizing the crucial role of gut microbiota in this process through the production of SCFAs and other functional metabolites. The steady decline in dietary fiber consumption over time has contributed to an imbalance in gut microbiota, negatively impacting human health and contributing to the global rise in diabetes, cancer, and other noncommunicable diseases [57]. The gut microbiome’s response to increased dietary fiber intake varies based on the type, amount, and duration of consumption, indicating the presence of fiber-specific threshold levels [58,59,60].

### 4.2. Dietary Proteins

In recent studies, participants following the high-protein diet (HPD) exhibited a marked expansion in microbial diversity as measured by the Shannon index, compared to those on the normal-protein diet (NPD). Additionally, the HPD led to notable differences in microbial composition after the intervention compared to the NPD. Both diets caused taxonomic shifts from baseline, including an enrichment of *Akkermansia* spp. and *Bifidobacterium* spp., along with a reduction in *Prevotella* spp. These findings demonstrate that weight-loss diets influence the gut microbiome in obesity and suggest that HPDs and NPDs have distinct effects, which may impact clinical responses to HPD [61,62].

Research on the microbiome has made substantial progress in understanding the microbial communities within the human body, utilizing techniques such as 16S rRNA gene sequencing and metagenomic analysis. Major projects like Meta HIT and the Human Microbiome Project (HMP) have generated extensive data on the diversity and genetic composition of the human microbiome. Dietary choices play a key role in shaping the gut microbiome, with a nutrient-rich diet promoting microbial diversity and beneficial bacteria, while frequent consumption of processed foods reduces diversity and encourages the growth of pathogenic species. These dietary differences significantly affect inflammation, metabolic health, and overall well-being [2,63,64].

### 4.3. Dietary Carbohydrates

Daily sugar intake should be limited to 5–8 g, yet the average American adolescent consumes approximately 41 g per day. This excessive consumption not only contributes to a higher (body mass index) BMI and an increased risk of diabetes, but also disrupts the gut microbiome. Research in rats has shown that early-life sugar consumption alters the gut microbiome independently of total caloric intake, body weight, or adiposity index, with these effects remaining consistent across various fructose-to-glucose ratios [65].

Diabetes has been associated with psychopathological distress in adolescents and is a growing health concern that can lead to long-term complications. Early onset of the disease presents significant challenges for both patients and healthcare professionals. Implementing dietary interventions, family psychotherapy, and nutritional education may play a crucial role in modifying unhealthy eating and drinking habits, ultimately contributing to improved health outcomes [44,57,66].

### 4.4. Alcohol Consumption

On the other hand, alcohol use can cause inflammation, disruption in the microbiome, and an increased risk of psychosomatic disorders. It can also lead to developmental issues in the nervous system, including disruption in reward pathways and addiction mechanisms, disruption in the normal development of the frontal lobe, with all the subsequent effects, as well as local bowel irritation that disrupts gut integrity [61]. Finally, alcohol intake has also been proven to reduce the population of healthy gut bacteria and increase the pro-inflammatory populations like *Proteobacteria* [67,68].

The relationship between diets and changes in gut composition are detailed explained in Table 1.

## 5. Adolescence: A Critical Stage in Development

Adolescence represents a critical period for the development of mental disorders, especially when we talk about those with inflammatory bowel diseases that started in childhood. A study from Sweden highlights an increased risk of anxiety, depression, and even suicide attempts in adolescents with inflammatory bowel diseases, suggesting an association between chronic inflammation, stress, and neuropsychiatric development. Genetic and environmental factors can trigger immunological and microbial changes, contributing to the development of intestinal inflammatory diseases [71,72,73,74].

Dysbiosis of the intestinal microbiota in childhood has been shown to influence neuropsychiatric development, thereby increasing the risk of neurodevelopmental disorders such as autism and ADHD. These intestinal imbalances influence both behavior and neuronal activity, underscoring the importance of maintaining a healthy microbiota during the critical period of adolescence [75].

**Figure 1:**
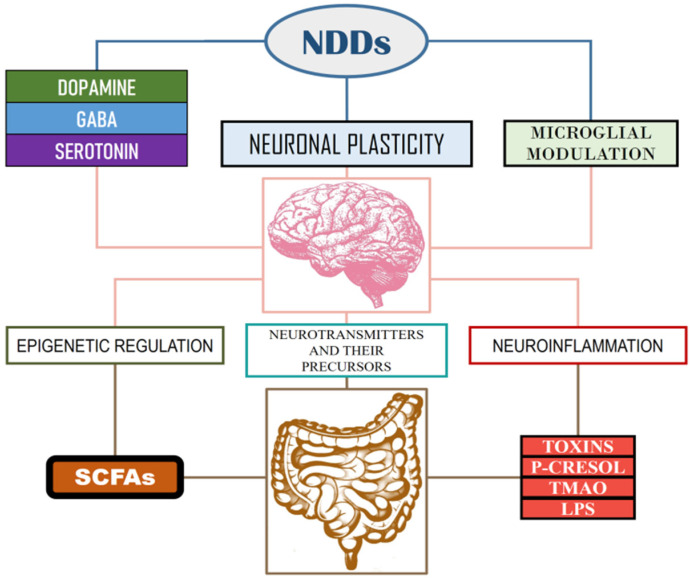
The influence of gut toxins, inflammation, and neurotransmitters on the brain development in adolescence [75].

Figure 1 illustrates how inflammation can influence brain development, while also affecting neuroplasticity and the normal functioning of the nervous system [75].

Also, another study highlights the association between dysbiosis and increased levels of systemic inflammation and neuroinflammation, also affecting essential neurotransmitters such as serotonin, dopamine, and gamma-aminobutyric acid, having a particularly important role in anxiety, depression, and other psychiatric disorders, with a high prevalence in adolescence [16].

In this critical period of development, the intestinal microbiota can directly affect cognitive and emotional processes through the gut–brain axis [76,77,78].

A healthy microbiome supports the normal development of the immune system and can positively impact mental health, thereby reducing the risk of mental disorders during adolescence [79,80].

At the same time, dysbiosis and inflammation simultaneously affect brain development and function, leading to the manifestation of gastrointestinal symptoms such as bloating, constipation, diarrhea and abdominal pain among children with autism-spectrum disorders [81,82].

## 6. The Link Between Microbiome, Diet, and Mental Health

Microbiota imbalances contribute to the emergence of disorders such as irritable bowel syndrome, which may coexist with stress-related mental health conditions. Modulating microbiota to improve mental health requires interventions such as probiotics, useful for improving the composition of gut microbiota, both in terms of improving gastrointestinal symptoms, as well as those related to anxiety and depression [83].

Mental health can be affected by the way the body responds to stress, which is influenced by the alteration of the intestinal microbiota. Gut bacteria can directly respond to stress-related signals, which highlight an interaction between the microbiome and the stress response [84].

**Figure 2:**
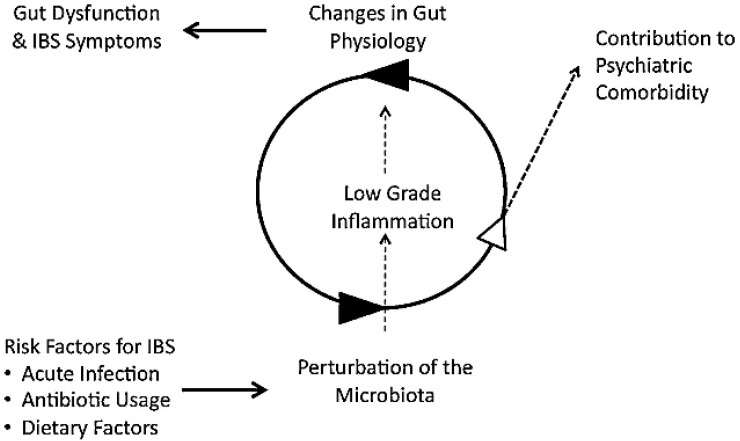
The role of food, acute infections, and antibiotic therapy as modulators on intestinal microbiota [85,86].

Food, acute infections, and antibiotic therapy play an essential role in modulating the microbiota. This translates into a low degree of inflammation, disruption of the gastrointestinal function, but also a series of psychiatric comorbidities (Figure 2) [85,86].

Studies have also identified significant differences in the composition of microbiota between children with ADHD and those without, suggesting a potential association between microbiota imbalances and ADHD manifestations. In children with ADHD, this is reflected in a lower microbial diversity, an abundance of pro-inflammatory bacteria, and a reduction in bacterial species involved in neurotransmitter synthesis [87,88].

Another study supports the connection between intestinal microbiota, diet, and mental health, highlighting that the use of prebiotics and probiotics was associated with an improvement in social behavior in children with autism spectrum disorders as well as an increase in Bifidobacteria populations. These findings suggest that microbiota modulation may have a positive impact on mental health. Additionally, the study emphasizes the role of probiotics, prebiotics, and diets rich in dairy products and omega-3 fatty acids in shaping the gut microbiota and influencing brain function [9,89,90,91,92]. Further explanations regarding the effects of dietary components on the gut microbiome and their impact on mental health are provided in Table 2.

Another study explores the correlation in patients diagnosed with anorexia nervosa. Upon hospital admission, these patients exhibited a reduced diversity of the gut microbiota compared to healthy controls. Low microbial diversity was associated with elevated levels of depression, anxiety, as well as symptoms characteristic of eating disorders. As nutritional status improved during treatment, both microbial diversity and psychiatric symptoms showed notable improvement [93]. In this pathology as well, a bidirectional interaction exists between the gastrointestinal tract and the central nervous system, known as the microbiota–gut–brain axis. This highlights the potential of modulating the gut microbiota as a strategy for the prevention and treatment of anorexia nervosa [94,95,96].

Regarding major depressive disorder, a study showed a decrease in *Faecalibacterium* in affected patients. *Faecalibacterium*, recognized for its anti-inflammatory properties, is significantly reduced in this group of patients, potentially contributing to systemic inflammation and the emergence of depressive symptoms [97,98].

Alterations in the gut microbiota can enhance the production of microbial lipopolysaccharides, which in turn may initiate inflammatory processes, contributing to the onset of depressive symptoms [99].

Critical developmental periods, such as intrauterine life and the early postnatal stages, have a significant impact on subsequent child behaviors. Alterations in the composition of gut microbiota during these stages can influence the child’s neurological development [100,101].

Exposure to stress during early life stages can disrupt the gut microbiota in children and has been linked to numerous long-term physical and mental health issues throughout childhood and adolescence. Chronic stress amplifies susceptibility to disease by enhancing vulnerability factors, including inflammation—a pathological process closely regulated by the gut microbiota [102,103,104,105,106].

Throughout the postnatal period and even into adulthood, the microbiota–gut–brain axis remains active, operating via neural, endocrine, and immunological pathways—among which intestinal permeability plays a particularly important role. Abnormal development of the brain may ultimately result in psychosis. Although the exact causes and mechanisms underlying psychosis remain unclear, neurodevelopmental disruption has been consistently documented and is considered a potential precursor. Numerous studies have identified a strong association between psychosis and inflammation. Given the close relationship between the gut microbiota and the immune system, it is hypothesized that microbiota may represent a key element in understanding the etiology and pathophysiology of psychosis [107].

It is particularly noteworthy that the initial colonization of the microbiome during fetal development occurs in parallel with the precisely timed and coordinated formation of the nervous system. Emerging research indicates that the microbiome actively contributes to the regulation of early brain development. However, disruptions to this delicate developmental process may adversely affect brain function, increasing the risk of neurodevelopmental and neuropsychiatric disorders such as autism spectrum disorder and attention deficit hyperactivity disorder [42].

## 7. Practical Implications and Recommendations

The human gut microbiota constitutes a highly intricate physiological system important for general health maintenance, immune function, metabolism, intestinal epithelial integrity, and cognitive processes. We can adopt a series of strategies for optimizing microbiome: increasing dietary fiber intake, incorporated fermented foods, reducing the intake of processed foods and added sugars, eating a diverse range of plant-based foods, consuming prebiotics foods, incorporating polyphenol-rich foods, and moderating alcohol consumption [108,109]. With these strategies, individuals can foster a healthy and divers microbiome, which is essential for overall health and well-being [110,111,112]. Also among the new strategies is supplementation with Vitamin D, Zinc, Folate, Omega-3, which benefit metabolic pathways, as the lack of these elements can lead to imbalances and health issues that are correlated with potential mental disorders [9,113,114,115,116]. Educational and intervention programs implemented in school and family settings are key strategies for optimizing dietary habits to support a healthy microbiome [117,118].

The development of eight Global Standards and 79 criteria for measuring them, namely (1) adolescents’ health literacy; (2) community support; (3) appropriate package of services; (4) providers’ competencies; (5) facility characteristics; (6) equity and nondiscrimination; (7) data and quality improvement; and (8) adolescents’ participation, can help to provide high-quality healthcare services for adolescents and guide them [119,120,121].

Nutrition-based interventions, which aim to maintain the balance of the microbiome, are relevant in the young population and extensively used. Interventions on food consumption show positive results with adolescent aggressive behaviors and some essential aspects of the interventions are the participation of parents and teachers [97,121].

## 8. Discussions

Based on the information provided by research conducted in preclinical and clinical areas, there are new emerging fields of interest regarding the link between gut microbiome and mental health, the physio-pathological mechanism of mental health, and new therapeutic perspectives. However, there are several limitations of the studies performed until this moment, with notable differences between preclinical and clinical research.

Regarding the gut–brain axis and mental health, some preclinical models demonstrated that germ-free mice displayed modified stress response [80], while others found that the transplantation of fecal microbiota from depressed patients to rodents induced depressive like behaviors [40]. Clinical studies support the theory that the gut microbiome influences mental health by altering metabolic pathways [11,96].

Some preclinical studies based on animal models support the hypothesis that changes in dietary habits modulate the composition of gut microbiota by influencing neurotransmitter synthesis [30,63]. Furthermore, clinical research on humans confirms the fact that different types of diets such as increased fiber intake improve depression and anxiety symptoms [58,62].

The impact of diets on adolescence period and neurodevelopment is demonstrated by preclinical and clinical research. Studies on rodents evidenced that early-life microbiota perturbation negatively affects neurodevelopment [42]. Clinical findings demonstrate that there is an alteration in microbiomes in teenagers with psychiatric pathologies [7,9].

Limitations in preclinical studies include the differences between studied species, as rodent models are not entirely representative of the human pathophysiology. Also, the composition of gut microbiome is different from rats to humans, while microbiota manipulation is artificial.

The sample size in clinical studies is usually small and not so heterogeneous as general populations, especially in adolescents. Most studies’ designs are observational and cross sectional and therefore cannot establish a reliable link between the factors analyzed and pathologies. Another limitation in clinical studies is represented by the lack of standardization of methods used in gut microbiota analysis.

## 9. Conclusions

The gut microbiome plays a vital role in both physical and mental health during childhood and adulthood. Gut microbiota begins developing at birth and stabilizes around age two. Adolescence is a sensitive period for brain development, during which disruptions in gut microbiota (dysbiosis) have a significant potential to influence mental health, contributing to onset on different disorders such as anxiety, depression, ADHD, and autism.

A balanced diet rich in fiber supports beneficial bacteria, which protects the gut microbiome and reduces chronic inflammation. High sugar consumption in the period of adolescence disturbs gut microbiome composition. Providing mental health support, especially to adolescents, may bring important changes with a protective role in developing chronic conditions such as diabetes, obesity, MDD, anxiety, ADHD and autism.

The gut–brain axis plays an indispensable role in the modulation of neurotransmitters (e.g., serotonin, dopamine, GABA, norepinephrine). The current literature data show that dysbiosis and chronic inflammation contribute to the development of psychiatric disorders in children and adolescents. Also, microbiota disruption is associated with conditions such as anorexia nervosa and major depressive disorder (MDD).

Adolescents with inflammatory bowel disease (IBD) have a higher risk of psychiatric disorders, including depression and suicidal behavior, due to chronic neuroinflammation. Inflammation in the gastrointestinal tract may also be linked to the onset of psychosis.

Long-term strategies such as improving diet, nutrient intake, limiting processed foods, avoiding alcohol and psychoactive substance consumption, and involving schools and families in health education and support are essential for supporting adolescents’ mental health. Implementing standardized or personalized care models and youth participation is essential to improve health services and outcomes for adolescents.

The current state of preclinical and clinical research on this topic is marked by several limitations: the differences between models based on rodents and human patho-physiology, the generally small human cohort studied, and the observational and cross-sectional study designs. Thus, in order to improve the quality of care in adolescents, much more research is needed in the near future, with a longitudinal study design and more age-appropriate samples of patients included in these studies. Also, the advances in microbiome profiling may allow for more personalized nutritional interventions.

## Figures and Tables

**Table 1:** Changes in gut composition in relation to food consumption [55,64,65,69,70].

Components	Structure	Effect
Protein	Increased protein intake	Increases beneficial *Lactobacillus* and *Bifidobacterium*; reduces harmful *Clostridium* and *Bacteroides*
	Increased animal-derived protein	Increases bile-tolerant anaerobes like *Bacteroides*, *Alistipes*, and *Bilophila*
	Increased total protein	Reduces certain beneficial bacteria and butyrate production; increases risk of IBD, higher levels of IGF-1: linked to cancer and diabetes risk
Fats	High-fat Western diets	Increases anaerobic microflora and *Bacteroides* counts
	Low-fat diet	Increases *Bifidobacterium*; reduces glucose and total cholesterol
	High saturated fat diet	Increases *Faecalibacterium prausnitzii*
	High monounsaturated fat intake	Reduces overall bacterial load and plasma cholesterol
Bioavailable carbohydrates	High glucose, fructose, and sucrose intake	Increases *Bifidobacteria*; reduces *Bacteroides*
	Supplementation with lactose	Reduces *Clostridia* species; increases beneficial SCFA concentration.
Non-digestible carbohydrates (fiber)	Probiotics-fermented food (cultured milk products, yogurt)	Increases total bacterial load and beneficial bacteria like *Bifidobacteria* and *Lactobacillus*; reduces enteropathogens like *Escherichia coli* and *Helicobacter pylori*
	Polyphenols	Increases *Bifidobacterium* and *Lactobacillus* and antibacterial activity against pathogens like *Staphylococcus aureus*, *Salmonella typhimurium*, pathogenic *Clostridium* species
	Prebiotics: soybeans, inulins, whole grains, oligosaccharides	Increases *Bifidobacteria*, lactic-acid bacteria, *Ruminococcus*, and *Eubacterium rectale*; reduces *Clostridium* and *Enterococcus*

**Table 2:** Effects of dietary components on gut microbiome and psychopathological mechanism [9,89,90,91,92].

Dietary Component	Effect on Gut and Microbiome	Psychopathological Mechanism
**Prebiotics** (e.g., inulin, FOS, GOS, XOS, COS)	↑ *Bifidobacterium* and *Lactobacillus* species	↑ SCAFs that reduce inflammation with a modulatory effect on gut–brain axis
**Probiotics** (e.g., *Lactobacillus* and *Bifidobacterium*)	↑ gut-barrier function ↑ SCAFs ↑ beneficial bacteria (*Bifidobacterium* and *Lactobacillus*) ↓ harmful species (e.g., Clostridium)	↑ synthesis of GABA, serotonin, and dopamine ↓ anxiety and depression ↓ cortisol level and stress mechanism
**Omega-3 fatty acids** (e.g., EPA and DHA)	↑ beneficial bacteria (*Bifidobacterium* and *Lactobacillus*) ↓ inflammatory cytokines (IL-1, IL-6, TNF-alfa) ↑ SCAFs	↑ neuroplasticity and cognitive function ↑ synthesis of serotonin and dopamine ↓ depressive symptoms Improves symptoms of autism-spectrum disorders
**Polyphenols**	↑ beneficial bacteria (*Bifidobacterium*, *Lactobacillus* and *Akkermansia*) ↑ SCAFs ↑ gut-barrier integrity ↓ harmful species (e.g., *Clostridium perfringens* and *Escherichia coli*) ↓ inflammation	↑ neuroplasticity and cognitive function ↑ synthesis of serotonin GABA and dopamine ↑ BDNF expression
**Dietary fibers**	↑ beneficial bacteria (*Bifidobacterium*, *Lactobacillus*, and *Faecalibacterium*) ↑ SCAFs ↓ production of endotoxins	↓ anxiety and depression ↑ BDNF ↑ synthesis of serotonin and dopamine

↑—increased; ↓—decreased.

## Data Availability

This study is based on previously published literature. No new data were generated in this literature review, and data sharing is not applicable.

## Abbreviations

The following abbreviations are used in this manuscript:

ADHD	Attention-Deficit Hyperactivity Disorder
BDNF	Brain-Derived Neurotrophic Factor
BMI	Body mass index
CNS	Central nervous system
COS	Chito-oligosaccharides
DHA	Docosahexaenoic Acid
EPA	Eicosapentaenoic Acid
FOS	Fructo-oligosaccharides
GABA	Gamma-aminobutyric acid
GBA	Gut–brain axis
GIT	Gastrointestinal tract
GOS	Galacto-oligosaccharides
HMP	Human Microbiome Project
HPA	Hypothalamic–pituitary–adrenal
HPD	High-protein diet
IBD	Intestinal bowel disease
IBS	Irritable bowel syndrome
IGF-1	Insulin-like growth factor 1
IL-1	Interleukin-1
IL-6	Interleukin-6
LPS	Lipopolysaccharides
MDD	Major depressive disorders
NA	Noradrenaline
NPD	Normal protein diet
rRNA	Ribosomal ribonucleic acid
SCFA	Short-chain fatty acid
SCFAs	Short-chain fatty acids
TMAO	Trimethylamine N-oxide
TNF-α	Tumor Necrosis Factor-alpha
VLCKD	Very low-calorie ketogenic diet
XOS	Xylo-oligosaccharides
